# AGE-VARIANT PATTERNS OF WITHIN-PERSON VARIATION IN SELF-REGULATORY CAPACITY REVEALED VIA AMBULATORY ASSESSMENTS

**DOI:** 10.1093/geroni/igad104.2044

**Published:** 2023-12-21

**Authors:** Jonathan Hakun, Daniel Elbich, Jessie Alwerdt, Lizbeth Benson, Allison Fleming

**Affiliations:** Pennsylvania State University, State College, Pennsylvania, United States; The Pennsylvania State University, Hershey, Pennsylvania, United States; Pennsylvania State University, University Park, Pennsylvania, United States; University of Oklahoma Health Sciences Center, Oklahoma City, Oklahoma, United States; Pennsylvania State University, University Park, Pennsylvania, United States

## Abstract

Stressful experiences often evoke changes in experienced negative affect. Individual differences in the degree of stressor-related reactivity may reflect the action of self-regulatory strategies that evolve over the adult lifespan. The executive hypothesis of self-regulation suggests that our ability to exert control over information processing (i.e. cognitive executive control) plays an important role in determining our effective capacity to regulate our emotions and behavior. We recently found evidence in younger adults that stressor reactivity is greater during moments when working memory capacity is lower than usual. Critically, aging is associated with normative declines in executive control and it remains unknown whether this impacts self-regulation in older adults. We’ll present the results of a recent ambulatory assessment study involving a sample of older adults (N = 80; 65-87 yrs; 79% female) where we sampled ratings of negative affect, exposure to everyday stressors, and working memory capacity up to 6 times per day for a period of 14 days. Results related to frequency of stressor exposures, mean levels of negative affect, and degree of negative affect reactivity to stressor exposures in this sample were largely consistent previous findings. However, when modeling stressor reactivity as a function of momentary variation in performance on ambulatory assessments of working memory capacity, we found evidence suggesting that older adults may react more to everyday stressors during moments with higher than average working memory capacity. We interpret these findings in terms of the limits of the executive hypothesis and other well-established theories of cognitive and psychological aging.

